# ID 350 - Efetividade Clínica e Custos do Emicizumabe para Hemofilia A: avaliação de desempenho pós-incorporação

**DOI:** 10.5327/2237-9622.2025.v34s1.350

**Published:** 2025-11-25

**Authors:** Juliana Alvares Teodoro, Ricardo Mesquita Camelo, Augusto Afonso Guerra, Luila Clicia Moura Henriques, Lívia Silva Nassif, Barbara Rodrigues Alvernaz dos Santos, Maria do Carmo da Silva Assunção, Eudes de Oliveira da Silveira, Luany Elvira Mesquita Carvalho, Tatyane Oliveira Rebouças, Melina Belintani Swain, Rafael Lucas de Assis Ferreira, Alexandra Vilela Gonçalves, Ieda Solange de Souza Pinto, Sandra Sibele de Figueiredo, Edilma Silva Casaretto, Ana Maria Vanderlei, Íris Maciel Costa, Neuza Cavalcanti de Morais Costa, Tânia Maria Rocha Guimarães, Melissa Palis Santana, Clarissa Barros Ferreira, Adriana Celia Luz, Francine Campoy, José Sávio Santos Ferreira, Andrea Aparecida Garcia, Barbara Cabrera, Paula Cella Giacometto, Carolina Freire da Gama Costa, Fátima Amaral Souto, Elaine Posener, Francisco de Assis Acurcio†

**Keywords:** hemofilia A, profilaxia, emicizumabe, sangramento, consumo, custos, EMCase

## Abstract

**Introdução:**

A hemofilia A (HA) é uma condição hereditária resultante de mutações no gene F8, que causam deficiência do fator de coagulação VIII (FVIII). O tratamento se baseia na reposição de FVIII e, atualmente, no uso de emicizumabe (EMI), anticorpo monoclonal biespecífico recomendado para profilaxia contra eventos hemorrágicos em pessoas com hemofilia A (PcHA). EMI foi incorporado pelo Sistema Único de Saúde (SUS) em 2019, e o objetivo deste trabalho foi avaliar a efetividade e os custos do EMI em relação à profilaxia utilizada anteriormente em PcHA.

**Método:**

O (EMCase) é um estudo observacional prospectivo que avalia PcHA em profilaxia com emicizumabe no Brasil, atendidos em hemocentros que integram o estudo. Foram analisados dados sociodemográficos e clínicos do período anterior (12 meses ou anualizado) e durante (12 meses ou anualizado) a profilaxia com EMI. Os sangramentos totais foram classificados em espontâneos, pós-traumáticos e de causa desconhecida, e contabilizados apenas se foram tratados. A taxa anualizada de sangramentos tratados (TAS) correspondeu ao somatório dos sangramentos tratados ao longo do tempo, transformando-se para um período de 365,25 dias (anualização). O consumo de produtos incluiu FVIII, agentes de (BpA) e EMI. O custo total do tratamento nos períodos pré e durante um ano de uso do EMI foi calculado considerando o valor pago pelo Ministério da Saúde, obtido do Banco de Preços da Saúde (BPS).

**Resultados:**

Foram incluídas 56 PcHA com pelo menos um ano de profilaxia com EMI. Desse total, 40 (71,4%) tinham HA grave e 47 (83,9%) apresentavam inibidor ao FVIII. A imunotolerância foi realizada por 41 (73,2%) PcHA. Antes de iniciar o EMI, 78,6% das PcHA estavam em profilaxia com FVIII e/ou BpA. A idade mediana no início do EMI foi 12,9 anos (1,9-71,0). A TAS total mediana reduziu de 3 (0-28) para 0 (0-3) (p<0,001). Não foram observadas diferenças significativas na TAS quando avaliados pacientes de maior gravidade ou com inibidores. O número de PcHA com zero sangramento aumentou de 21,4% para 76,8% (p=0,027). Antes da profilaxia com EMI, o consumo/kg mensal médio e o custo/kg mensal foram de: FVIIIr 106,6 UI/kg/mês e R$ 2.567,20 por kg/mês; FVIIIdp 2,5 UI/kg/mês e R$ 44,45 por kg/mês; complexo protrombínico parcialmente ativado (CCPa) 436,2 UI/kg/mês e R$ 30.533,80 por kg/mês; fator VII ativado recombinante (rFVIIa) 4,5 mg/kg/mês e R$ 12.476,02 por kg/mês. O custo total antes do EMI foi de R$ 45.621,47. Durante a profilaxia com EMI, uma PcHA recebeu CCPa (9,7 UI/kg/mês e R$ 12,16 por kg/mês), 46,4% receberam rFVIIa (1,0 mg/kg/mês e R$48,47 por kg/mês) e 12,5% receberam FVIIIr (314,7 UI/kg/mês e R$ 135,31 por kg/mês). O consumo total de EMI no primeiro ano de profilaxia foi 236.042,0 mg, resultando em consumo/kg mensal mediano de 7,1 mg/kg/mês e custo/kg mensal foi de R$ 40.029,35 por kg/mês. O custo total durante o uso de EMI foi de R$ 40.225,29. Houve redução de 11,8% nos custos/kg mensais, quando comparados os períodos pré e durante EMI.

**Conclusão:**

Evidências científicas demonstram que a profilaxia com EMI está associada à redução de sangramentos e ao menor consumo de produtos em PcHA. Neste estudo, o uso de EMI resultou em uma diminuição da TAS total, do número de episódios de sangramento, do consumo de FVIII e agentes de BpA e dos custos totais de tratamento quando comparado à profilaxia anterior.

